# *catena*-Poly[[[(acetato-κ^2^ *O*,*O*′)cadmium]-μ-acetato-κ^3^ *O*,*O*′:*O*′-μ-{1,2-bis­[4-(pyridin-3-yl)pyrimidin-2-ylsulfan­yl]ethane}-κ^2^ *N* ^4^,*N* ^4′^] trihydrate]

**DOI:** 10.1107/S1600536811046794

**Published:** 2011-11-12

**Authors:** Hua-Ze Dong, Zhao-Lian Chu

**Affiliations:** a Deparment of Chemistry and Chemical Engineering, Hefei Normal University, Hefei 230061, People’s Republic of China; b School of Chemistry and Chemical Engineering, Anhui University of Technology, Maanshan 243002, People’s Republic of China

## Abstract

The title compound, {[Cd(CH_3_COO)_2_(C_20_H_16_N_6_S_2_)]·3H_2_O}_*n*_, exists as a one-dimensional zigzag polymer in which the Cd^II^ ion shows a seven-coordinate [CdO_5_N_2_] distorted penta­gonal–bipyramidal geometry with the N atoms in axial positions and an N—Cd—N angle of 176.94 (13)°. The metal atoms are bridged by 1,2-bis­[4-(pyridin-3-yl)pyrimidin-2-ylsulfan­yl]ethane ligands, giving a polymeric chain extending along the *b* axis. Adjacent chains related by an inversion center are further bridged by Cd—O bonds formed between the O atom of one of the acetate ligands and the metal atom. The five Cd—O bond lengths are in the range 2.329 (3)–2.485 (3) Å. There are π–π stacking inter­actions between the aromatic rings of adjacent polymeric chains, the centroid–centroid distances being 3.556 (3) and 3.698 (3) Å, organizing the chains into a three-dimensional framework. This framework is additionally stabilized by extensive O—H⋯O and O—H⋯N hydrogen bonding between water mol­ecules and the ligands.

## Related literature

For backgroud to coordination polymers with thio­ether ligands, see: Dong, Yang *et al.* (2008 ▶); Dong, Zhu *et al.* (2008 ▶); Dong *et al.* (2009 ▶). 
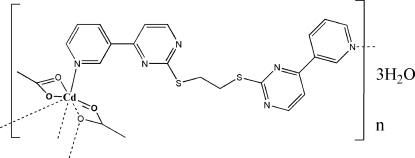


## Experimental

### 

#### Crystal data

[Cd(C_2_H_3_O_2_)_2_(C_20_H_16_N_6_S_2_)]·3H_2_O
*M* *_r_* = 689.07
Monoclinic, 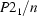

*a* = 10.8000 (12) Å
*b* = 14.0816 (16) Å
*c* = 18.594 (2) Å
β = 95.997 (2)°
*V* = 2812.4 (5) Å^3^
*Z* = 4
Mo *K*α radiation
μ = 0.98 mm^−1^
*T* = 291 K
0.20 × 0.10 × 0.03 mm

#### Data collection

Bruker SMART CCD area-detector diffractometer
Absorption correction: multi-scan (*SADABS*; Bruker, 2000 ▶) *T*_min_ = 0.822, *T*_max_ = 0.971
14681 measured reflections
5492 independent reflections
3846 reflections with *I* > 2σ(*I*)
*R*_int_ = 0.048

#### Refinement

*R*[*F* ^2^ > 2σ(*F* ^2^)] = 0.054
*wR*(*F* ^2^) = 0.112
*S* = 1.01
5492 reflections
363 parameters
H-atom parameters constrained
Δρ_max_ = 0.84 e Å^−3^
Δρ_min_ = −0.77 e Å^−3^

### 

Data collection: *SMART* (Bruker, 2000 ▶); cell refinement: *SAINT* (Bruker, 2000 ▶); data reduction: *SAINT*; program(s) used to solve structure: *SHELXTL* (Sheldrick, 2008 ▶); program(s) used to refine structure: *SHELXTL*; molecular graphics: *SHELXTL*; software used to prepare material for publication: *SHELXTL*.

## Supplementary Material

Crystal structure: contains datablock(s) I, global. DOI: 10.1107/S1600536811046794/gk2423sup1.cif

Structure factors: contains datablock(s) I. DOI: 10.1107/S1600536811046794/gk2423Isup2.hkl

Additional supplementary materials:  crystallographic information; 3D view; checkCIF report

## Figures and Tables

**Table 1:** Hydrogen-bond geometry (Å, °)

*D*—H⋯*A*	*D*—H	H⋯*A*	*D*⋯*A*	*D*—H⋯*A*
O6—H6*OB*⋯O1^i^	0.87	2.24	3.102 (6)	167
O7—H7*OB*⋯N2^ii^	0.85	2.15	3.000 (5)	173
O7—H7*OA*⋯O4^iii^	0.85	2.00	2.819 (6)	162
O5—H5*A*⋯O6	0.85	2.01	2.782 (5)	150
O5—H5*B*⋯O2^iv^	0.85	2.02	2.864 (5)	177

Symmetry codes: (i) 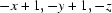; (ii) 
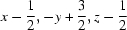
; (iii) 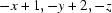; (iv) 
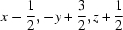
.

**Fig. 1.**
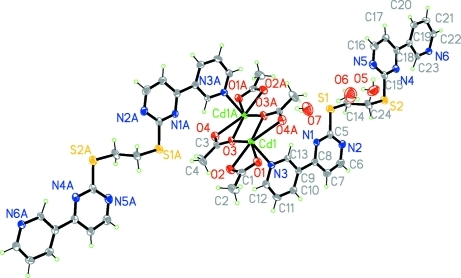
The structure of the title compound showing 30% probability displacement ellipsoids. Atoms with the label A are related to the labelled atoms by the symmetry operation -x,1 - y, 1 - z.

**Fig. 2.**
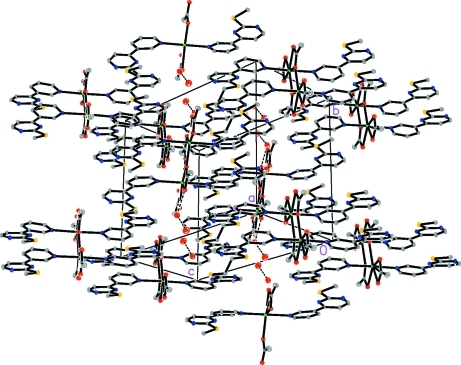
Perspective view of intermolecular hydrogen bonding and π–π stacking interactions in the title complex

## supplementary crystallographic information

### Comment

In recent years flexible thioethers have been extensively studied as they are capable of displaying different reactions in various circumstances, giving rise to metal-organic frameworks with fascinating structures (Dong *et al.*, 2009; Dong,Yang *et al.*, 2008; Dong, Zhu *et al.*, 2008). Here we report the structure of the title one-dimensional cadmium(II) complex, [*L*Cd(CH_3_COO)_2_(H_2_O)_3_]_n_.

The title compound exists as a one-dimensional zigzag polymer, with seven-coordinate CdO_5_N_2_ core, in which Cd^II^ centers are bridged by the organic ligand to give polymeric chain, and two oxygen atoms from two anions bridge two adjacent polymeric chains as shown in Figure 1. The axial N3—Cd—N6 bond angle is 176.91 (14)°. There are π–π stacking interactions between the aromatic rings from adjacent polymeric chains with the centroid-centroid distances of 3.556 and 3.698 Å, organizing the chains into a three-dimensional framework. This framework is additionally stabilized by numerous hydrogen bonds between water molecules and the ligands (Fig. 2) .

### Experimental

All solvents and chemicals were of analytical grade and were used without further purification. The title compound was prepared by similar procedure to that reported in the literature (Dong *et al.*, 2009). The complex was synthesized by adding methanol (25 ml) solution of ligand (40.4 mg, 1.0 mmol) to a stirred methanol (20 ml) solution of Cd(OAc)_2_2H_2_O (26.6 mg, 1.0 mmol). The resulting solution was refluxed for one hour, cooled to room temperature, filtered, and evaporated slowly to give the colorless single crystals. IR (KBr, cm^-1^): 3417 (m), 1561 (s), 1480 (w), 1413 (m), 1339 (m), 1325 (m), 1225 (w), 1190 (m), 1028 (w), 807 (w).

### Refinement

Hydrogen atoms from the L ligand were geomemetrically positioned (C—H 0.93–0.97 Å) and refined as riding, with *U*_iso_(H)=1.2–1.5 *U*_eq_ of the parent atom. Positions of the water molecules H atoms were calculated geometrically assuming their involvement in hydrogen bonding and refined as riding with *U*_iso_(H)=1.5 *U*_eq_(O) .

### Crystal data

**Table:**

[Cd(C_2_H_3_O_2_)_2_(C_20_H_16_N_6_S_2_)]·3H_2_O	*F*(000) = 1400
*M**_r_* = 689	*D*_x_ = 1.628 Mg m^−^^3^
Monoclinic, *P*2_1_/*n*	Mo *K*α radiation, λ = 0.71073 Å
Hall symbol: -P 2yn	Cell parameters from 3846 reflections
*a* = 10.8000 (12) Å	θ = 1.8–25.2°
*b* = 14.0816 (16) Å	µ = 0.98 mm^−^^1^
*c* = 18.594 (2) Å	*T* = 291 K
β = 95.997 (2)°	Plate, colorless
*V* = 2812.4 (5) Å^3^	0.20 × 0.10 × 0.03 mm
*Z* = 4	

### Data collection

**Table:**

Bruker SMART CCD area-detector diffractometer	5492 independent reflections
Radiation source: fine-focus sealed tube	3846 reflections with *I* > 2σ(*I*)
graphite	*R*_int_ = 0.048
φ and ω scan	θ_max_ = 26.0°, θ_min_ = 1.8°
Absorption correction: multi-scan (*SADABS*; Bruker, 2000)	*h* = −7→13
*T*_min_ = 0.822, *T*_max_ = 0.971	*k* = −16→17
14681 measured reflections	*l* = −22→22

### Refinement

**Table:**

Refinement on *F*^2^	Primary atom site location: structure-invariant direct methods
Least-squares matrix: full	Secondary atom site location: difference Fourier map
*R*[*F*^2^ > 2σ(*F*^2^)] = 0.054	Hydrogen site location: inferred from neighbouring sites
*wR*(*F*^2^) = 0.112	H-atom parameters constrained
*S* = 1.01	*w* = 1/[σ^2^(*F*_o_^2^) + (0.0473*P*)^2^] where *P* = (*F*_o_^2^ + 2*F*_c_^2^)/3
5492 reflections	(Δ/σ)_max_ = 0.005
363 parameters	Δρ_max_ = 0.84 e Å^−^^3^
0 restraints	Δρ_min_ = −0.77 e Å^−^^3^

### Special details

**Table:**

Geometry. All esds (except the esd in the dihedral angle between two l.s. planes) are estimated using the full covariance matrix. The cell esds are taken into account individually in the estimation of esds in distances, angles and torsion angles; correlations between esds in cell parameters are only used when they are defined by crystal symmetry. An approximate (isotropic) treatment of cell esds is used for estimating esds involving l.s. planes.
Refinement. Refinement of F^2^ against ALL reflections. The weighted R-factor wR and goodness of fit S are based on F^2^, conventional R-factors R are based on F, with F set to zero for negative F^2^. The threshold expression of F^2^ > 2sigma(F^2^) is used only for calculating R-factors(gt) etc. and is not relevant to the choice of reflections for refinement. R-factors based on F^2^ are statistically about twice as large as those based on F, and R- factors based on ALL data will be even larger.

### Fractional atomic coordinates and isotropic or equivalent isotropic displacement parameters (Å^2^)

**Table:**

	*x*	*y*	*z*	*U*_iso_*/*U*_eq_	
C1	0.6658 (4)	0.8014 (4)	−0.1567 (3)	0.0429 (13)	
C2	0.7246 (5)	0.7596 (4)	−0.2185 (3)	0.0683 (18)	
H2A	0.8133	0.7580	−0.2071	0.103*	
H2B	0.7041	0.7976	−0.2609	0.103*	
H2C	0.6941	0.6961	−0.2273	0.103*	
C3	0.5891 (4)	1.1395 (4)	−0.0653 (2)	0.0363 (12)	
C4	0.6550 (6)	1.1502 (5)	−0.1300 (3)	0.075 (2)	
H4A	0.6646	1.2165	−0.1402	0.112*	
H4B	0.6078	1.1202	−0.1704	0.112*	
H4C	0.7355	1.1210	−0.1218	0.112*	
C5	0.6748 (5)	0.8859 (3)	0.3150 (2)	0.0389 (12)	
C6	0.8781 (6)	0.8792 (4)	0.3511 (3)	0.0592 (16)	
H6	0.9437	0.8767	0.3876	0.071*	
C7	0.9059 (5)	0.8780 (4)	0.2821 (3)	0.0532 (15)	
H7	0.9879	0.8752	0.2713	0.064*	
C8	0.8070 (5)	0.8813 (3)	0.2277 (2)	0.0363 (11)	
C9	0.8240 (4)	0.8842 (3)	0.1498 (2)	0.0343 (11)	
C10	0.9383 (5)	0.8957 (3)	0.1249 (3)	0.0426 (12)	
H10	1.0103	0.8985	0.1570	0.051*	
C11	0.9446 (5)	0.9032 (4)	0.0512 (3)	0.0472 (14)	
H11	1.0212	0.9099	0.0331	0.057*	
C12	0.8387 (5)	0.9007 (3)	0.0058 (3)	0.0417 (13)	
H12	0.8441	0.9079	−0.0435	0.050*	
C13	0.7214 (4)	0.8809 (3)	0.0997 (2)	0.0342 (11)	
H13	0.6438	0.8731	0.1163	0.041*	
C14	0.5271 (5)	0.9099 (4)	0.4283 (2)	0.0456 (14)	
H14A	0.5958	0.9520	0.4439	0.055*	
H14B	0.4511	0.9399	0.4404	0.055*	
H24A	0.4748	0.7758	0.4556	0.055*	
H24B	0.6195	0.7861	0.4566	0.055*	
C15	0.4022 (5)	0.8463 (3)	0.5830 (2)	0.0393 (12)	
C16	0.1985 (5)	0.8570 (4)	0.5474 (3)	0.0536 (15)	
H16	0.1324	0.8566	0.5111	0.064*	
C17	0.1719 (5)	0.8667 (3)	0.6172 (3)	0.0470 (14)	
H17	0.0907	0.8746	0.6286	0.056*	
C18	0.2728 (5)	0.8643 (3)	0.6708 (2)	0.0349 (12)	
C19	0.2573 (5)	0.8719 (3)	0.7486 (2)	0.0357 (12)	
C20	0.1445 (5)	0.8667 (3)	0.7764 (3)	0.0393 (13)	
H20	0.0713	0.8598	0.7457	0.047*	
C21	0.1409 (5)	0.8719 (3)	0.8492 (3)	0.0447 (13)	
H21	0.0650	0.8688	0.8685	0.054*	
C22	0.2486 (5)	0.8814 (3)	0.8939 (3)	0.0397 (12)	
H22	0.2441	0.8834	0.9436	0.048*	
C23	0.3635 (5)	0.8826 (3)	0.7981 (2)	0.0367 (11)	
H23	0.4407	0.8862	0.7802	0.044*	
C24	0.5447 (5)	0.8173 (4)	0.4694 (3)	0.0509 (15)	
Cd1	0.54604 (3)	0.88993 (3)	−0.053496 (16)	0.03387 (13)	
N1	0.6904 (4)	0.8839 (3)	0.24501 (19)	0.0355 (9)	
N2	0.7636 (4)	0.8836 (3)	0.3705 (2)	0.0456 (11)	
N3	0.7268 (4)	0.8883 (3)	0.0283 (2)	0.0384 (9)	
N4	0.3887 (4)	0.8531 (3)	0.6531 (2)	0.0395 (10)	
N5	0.3119 (4)	0.8480 (3)	0.5283 (2)	0.0511 (12)	
N6	0.3595 (4)	0.8880 (3)	0.86954 (19)	0.0362 (9)	
O1	0.6169 (3)	0.7495 (2)	−0.11385 (17)	0.0490 (9)	
O2	0.6649 (3)	0.8906 (3)	−0.15103 (17)	0.0493 (9)	
O3	0.5654 (3)	1.0578 (2)	−0.04314 (15)	0.0430 (8)	
O4	0.5594 (3)	1.2110 (3)	−0.03123 (17)	0.0508 (9)	
O5	0.3481 (4)	0.5134 (3)	0.2749 (2)	0.0912 (15)	
H5A	0.3204	0.5168	0.2305	0.137*	
H5B	0.2928	0.5397	0.2974	0.137*	
O6	0.3059 (5)	0.4610 (4)	0.1302 (2)	0.1181 (18)	
H6OA	0.3320	0.5000	0.1002	0.177*	
H6OB	0.3213	0.4025	0.1184	0.177*	
O7	0.3543 (4)	0.6001 (3)	0.0280 (2)	0.0916 (15)	
H7OB	0.3312	0.5997	−0.0173	0.137*	
H7OA	0.3742	0.6575	0.0380	0.137*	
S1	0.51853 (13)	0.89534 (11)	0.33124 (7)	0.0523 (4)	
S2	0.55806 (13)	0.83446 (11)	0.56618 (7)	0.0501 (4)	

### Atomic displacement parameters (Å^2^)

**Table:**

	*U*^11^	*U*^22^	*U*^33^	*U*^12^	*U*^13^	*U*^23^
C1	0.032 (3)	0.067 (4)	0.030 (3)	0.008 (3)	0.001 (2)	−0.010 (3)
C2	0.075 (5)	0.072 (5)	0.064 (4)	−0.003 (3)	0.034 (3)	−0.019 (3)
C3	0.035 (3)	0.049 (4)	0.024 (2)	−0.008 (2)	0.003 (2)	0.004 (2)
C4	0.095 (5)	0.095 (5)	0.039 (3)	−0.021 (4)	0.029 (3)	−0.002 (3)
C5	0.048 (3)	0.033 (3)	0.035 (3)	−0.004 (2)	0.001 (2)	0.004 (2)
C6	0.054 (4)	0.083 (5)	0.037 (3)	0.013 (3)	−0.011 (3)	−0.001 (3)
C7	0.041 (3)	0.079 (4)	0.039 (3)	0.009 (3)	0.000 (2)	0.004 (3)
C8	0.044 (3)	0.029 (3)	0.036 (3)	0.001 (2)	0.005 (2)	0.003 (2)
C9	0.036 (3)	0.029 (3)	0.038 (3)	0.001 (2)	0.003 (2)	0.003 (2)
C10	0.036 (3)	0.046 (3)	0.044 (3)	0.001 (3)	−0.003 (2)	0.000 (3)
C11	0.033 (3)	0.062 (4)	0.048 (3)	−0.005 (3)	0.012 (2)	0.002 (3)
C12	0.049 (3)	0.046 (3)	0.032 (3)	−0.003 (3)	0.010 (2)	0.000 (2)
C13	0.031 (3)	0.039 (3)	0.033 (3)	−0.001 (2)	0.005 (2)	0.007 (2)
C14	0.056 (4)	0.051 (4)	0.031 (3)	0.000 (3)	0.012 (2)	−0.004 (2)
C15	0.054 (4)	0.035 (3)	0.030 (3)	−0.010 (2)	0.005 (2)	0.000 (2)
C16	0.049 (4)	0.070 (4)	0.038 (3)	−0.016 (3)	−0.011 (3)	0.001 (3)
C17	0.044 (3)	0.061 (4)	0.034 (3)	−0.007 (3)	−0.006 (2)	0.006 (2)
C18	0.045 (3)	0.027 (3)	0.032 (3)	−0.004 (2)	0.001 (2)	−0.0037 (19)
C19	0.045 (3)	0.029 (3)	0.032 (2)	−0.001 (2)	−0.002 (2)	−0.003 (2)
C20	0.035 (3)	0.037 (3)	0.044 (3)	−0.005 (2)	−0.002 (2)	0.000 (2)
C21	0.040 (3)	0.049 (4)	0.047 (3)	−0.007 (2)	0.011 (3)	−0.004 (2)
C22	0.048 (3)	0.041 (3)	0.031 (3)	−0.002 (3)	0.006 (2)	−0.001 (2)
C23	0.042 (3)	0.037 (3)	0.032 (3)	0.004 (2)	0.012 (2)	0.007 (2)
C24	0.076 (4)	0.046 (4)	0.034 (3)	−0.010 (3)	0.022 (3)	−0.005 (2)
Cd1	0.0364 (2)	0.0440 (2)	0.02189 (17)	−0.00100 (18)	0.00639 (13)	−0.00316 (16)
N1	0.035 (3)	0.042 (2)	0.029 (2)	−0.002 (2)	−0.0005 (17)	0.0064 (18)
N2	0.049 (3)	0.057 (3)	0.030 (2)	0.005 (2)	−0.002 (2)	0.003 (2)
N3	0.038 (3)	0.043 (2)	0.035 (2)	0.000 (2)	0.0084 (18)	0.0018 (19)
N4	0.050 (3)	0.039 (3)	0.030 (2)	−0.005 (2)	0.003 (2)	−0.0017 (18)
N5	0.054 (3)	0.065 (3)	0.032 (2)	−0.015 (2)	−0.005 (2)	0.002 (2)
N6	0.039 (3)	0.040 (2)	0.030 (2)	0.001 (2)	0.0054 (18)	−0.0010 (19)
O1	0.056 (3)	0.058 (3)	0.0340 (19)	−0.0056 (18)	0.0105 (17)	−0.0006 (17)
O2	0.061 (3)	0.050 (2)	0.041 (2)	0.000 (2)	0.0203 (17)	−0.0083 (18)
O3	0.057 (2)	0.039 (2)	0.0349 (19)	−0.0042 (17)	0.0126 (16)	0.0034 (15)
O4	0.065 (3)	0.042 (2)	0.046 (2)	−0.0009 (18)	0.0081 (18)	−0.0023 (17)
O5	0.076 (3)	0.116 (4)	0.081 (3)	0.039 (3)	0.008 (2)	0.000 (3)
O6	0.151 (5)	0.115 (5)	0.090 (4)	0.020 (4)	0.022 (3)	0.005 (3)
O7	0.112 (4)	0.099 (4)	0.062 (3)	−0.025 (3)	0.006 (3)	0.004 (2)
S1	0.0456 (9)	0.0780 (11)	0.0339 (7)	−0.0040 (8)	0.0078 (6)	0.0002 (7)
S2	0.0574 (10)	0.0584 (9)	0.0354 (7)	−0.0007 (7)	0.0094 (6)	0.0021 (6)

### Geometric parameters (Å, °)

**Table:**

C1—O1	1.239 (6)	C15—S2	1.752 (5)
C1—O2	1.260 (6)	C16—N5	1.317 (6)
C1—C2	1.492 (6)	C16—C17	1.365 (7)
C2—H2A	0.9600	C16—H16	0.9300
C2—H2B	0.9600	C17—C18	1.398 (6)
C2—H2C	0.9600	C17—H17	0.9300
C3—O4	1.249 (6)	C18—N4	1.336 (6)
C3—O3	1.259 (6)	C18—C19	1.478 (6)
C3—C4	1.468 (7)	C19—C20	1.373 (7)
C4—H4A	0.9600	C19—C23	1.401 (6)
C4—H4B	0.9600	C20—C21	1.361 (6)
C4—H4C	0.9600	C20—H20	0.9300
C5—N1	1.330 (5)	C21—C22	1.363 (6)
C5—N2	1.333 (6)	C21—H21	0.9300
C5—S1	1.750 (5)	C22—N6	1.328 (6)
C6—N2	1.324 (7)	C22—H22	0.9300
C6—C7	1.347 (7)	C23—N6	1.336 (5)
C6—H6	0.9300	C23—H23	0.9300
C7—C8	1.393 (6)	C24—S2	1.806 (5)
C7—H7	0.9300	C24—H24A	0.9679
C8—N1	1.333 (6)	C24—H24B	0.9703
C8—C9	1.480 (6)	Cd1—O2	2.329 (3)
C9—C13	1.372 (6)	Cd1—N3	2.346 (4)
C9—C10	1.373 (6)	Cd1—N6^i^	2.346 (4)
C10—C11	1.382 (6)	Cd1—O3	2.378 (3)
C10—H10	0.9300	Cd1—O3^ii^	2.382 (3)
C11—C12	1.350 (6)	Cd1—O1	2.437 (3)
C11—H11	0.9300	Cd1—O4^ii^	2.485 (3)
C12—N3	1.331 (6)	N6—Cd1^iii^	2.346 (4)
C12—H12	0.9300	O3—Cd1^ii^	2.382 (3)
C13—N3	1.340 (5)	O4—Cd1^ii^	2.485 (3)
C13—H13	0.9300	O5—H5A	0.8500
C14—C24	1.514 (7)	O5—H5B	0.8500
C14—S1	1.810 (4)	O6—H6OA	0.8520
C14—H14A	0.9700	O6—H6OB	0.8735
C14—H14B	0.9700	O7—H7OB	0.8519
C15—N4	1.329 (5)	O7—H7OA	0.8528
C15—N5	1.334 (6)		
			
O1—C1—O2	121.8 (5)	C20—C19—C23	117.2 (4)
O1—C1—C2	120.4 (5)	C20—C19—C18	124.0 (4)
O2—C1—C2	117.8 (5)	C23—C19—C18	118.7 (5)
C1—C2—H2A	109.5	C21—C20—C19	119.3 (5)
C1—C2—H2B	109.5	C21—C20—H20	120.4
H2A—C2—H2B	109.5	C19—C20—H20	120.4
C1—C2—H2C	109.5	C20—C21—C22	120.1 (5)
H2A—C2—H2C	109.5	C20—C21—H21	120.0
H2B—C2—H2C	109.5	C22—C21—H21	120.0
O4—C3—O3	119.9 (4)	N6—C22—C21	122.8 (4)
O4—C3—C4	120.4 (5)	N6—C22—H22	118.6
O3—C3—C4	119.7 (5)	C21—C22—H22	118.6
C3—C4—H4A	109.5	N6—C23—C19	123.3 (4)
C3—C4—H4B	109.5	N6—C23—H23	118.3
H4A—C4—H4B	109.5	C19—C23—H23	118.3
C3—C4—H4C	109.5	C14—C24—S2	112.3 (4)
H4A—C4—H4C	109.5	C14—C24—H24A	109.4
H4B—C4—H4C	109.5	S2—C24—H24A	108.9
N1—C5—N2	127.0 (5)	C14—C24—H24B	109.2
N1—C5—S1	113.2 (3)	S2—C24—H24B	108.9
N2—C5—S1	119.9 (4)	H24A—C24—H24B	108.0
N2—C6—C7	124.5 (5)	O2—Cd1—N3	90.92 (13)
N2—C6—H6	117.7	O2—Cd1—N6^i^	91.89 (12)
C7—C6—H6	117.7	N3—Cd1—N6^i^	176.94 (13)
C6—C7—C8	117.4 (5)	O2—Cd1—O3	90.46 (12)
C6—C7—H7	121.3	N3—Cd1—O3	84.09 (12)
C8—C7—H7	121.3	N6^i^—Cd1—O3	97.09 (12)
N1—C8—C7	119.8 (4)	O2—Cd1—O3^ii^	161.70 (12)
N1—C8—C9	116.9 (4)	N3—Cd1—O3^ii^	88.04 (12)
C7—C8—C9	123.2 (5)	N6^i^—Cd1—O3^ii^	89.68 (12)
C13—C9—C10	117.8 (4)	O3—Cd1—O3^ii^	71.26 (12)
C13—C9—C8	119.3 (4)	O2—Cd1—O1	54.50 (11)
C10—C9—C8	122.8 (4)	N3—Cd1—O1	90.60 (12)
C9—C10—C11	118.8 (4)	N6^i^—Cd1—O1	90.04 (12)
C9—C10—H10	120.6	O3—Cd1—O1	144.54 (11)
C11—C10—H10	120.6	O3^ii^—Cd1—O1	143.75 (11)
C12—C11—C10	119.5 (5)	O2—Cd1—O4^ii^	145.35 (12)
C12—C11—H11	120.3	N3—Cd1—O4^ii^	89.36 (12)
C10—C11—H11	120.3	N6^i^—Cd1—O4^ii^	87.65 (12)
N3—C12—C11	123.0 (5)	O3—Cd1—O4^ii^	124.00 (11)
N3—C12—H12	118.5	O3^ii^—Cd1—O4^ii^	52.93 (12)
C11—C12—H12	118.5	O1—Cd1—O4^ii^	90.85 (12)
N3—C13—C9	123.7 (4)	C5—N1—C8	117.2 (4)
N3—C13—H13	118.1	C6—N2—C5	114.1 (4)
C9—C13—H13	118.1	C12—N3—C13	117.1 (4)
C24—C14—S1	113.4 (4)	C12—N3—Cd1	121.1 (3)
C24—C14—H14A	108.9	C13—N3—Cd1	121.6 (3)
S1—C14—H14A	108.9	C15—N4—C18	116.9 (4)
C24—C14—H14B	108.9	C16—N5—C15	114.9 (4)
S1—C14—H14B	108.9	C22—N6—C23	117.3 (4)
H14A—C14—H14B	107.7	C22—N6—Cd1^iii^	122.7 (3)
N4—C15—N5	126.9 (5)	C23—N6—Cd1^iii^	119.5 (3)
N4—C15—S2	112.8 (4)	C1—O1—Cd1	89.5 (3)
N5—C15—S2	120.3 (4)	C1—O2—Cd1	94.0 (3)
N5—C16—C17	124.1 (5)	C3—O3—Cd1	154.4 (3)
N5—C16—H16	118.0	C3—O3—Cd1^ii^	95.8 (3)
C17—C16—H16	118.0	Cd1—O3—Cd1^ii^	108.74 (12)
C16—C17—C18	116.7 (5)	C3—O4—Cd1^ii^	91.2 (3)
C16—C17—H17	121.6	H5A—O5—H5B	105.1
C18—C17—H17	121.6	H6OA—O6—H6OB	111.0
N4—C18—C17	120.5 (5)	H7OB—O7—H7OA	105.3
N4—C18—C19	117.1 (4)	C5—S1—C14	103.4 (2)
C17—C18—C19	122.4 (5)	C15—S2—C24	102.3 (2)

Symmetry codes: (i) *x*, *y*, *z*−1; (ii) −*x*+1, −*y*+2, −*z*; (iii) *x*, *y*, *z*+1.

### Hydrogen-bond geometry (Å, °)

**Table:**

*D*—H···*A*	*D*—H	H···*A*	*D*···*A*	*D*—H···*A*
O6—H6OB···O1^iv^	0.87	2.24	3.102 (6)	167
O7—H7OB···N2^v^	0.85	2.15	3.000 (5)	173
O7—H7OA···O4^ii^	0.85	2.00	2.819 (6)	162
O5—H5A···O6	0.85	2.01	2.782 (5)	150
O5—H5B···O2^vi^	0.85	2.02	2.864 (5)	177
C6—H6···O1^vii^	0.93	2.58	3.163 (7)	121
C10—H10···O5^viii^	0.93	2.48	3.263 (7)	142

Symmetry codes: (iv) −*x*+1, −*y*+1, −*z*; (v) *x*−1/2, −*y*+3/2, *z*−1/2; (ii) −*x*+1, −*y*+2, −*z*; (vi) *x*−1/2, −*y*+3/2, *z*+1/2; (vii) *x*+1/2, −*y*+3/2, *z*+1/2; (viii) −*x*+3/2, *y*+1/2, −*z*+1/2.
